# *Pichia fabianii* blood infection in a premature infant in China: case report

**DOI:** 10.1186/1756-0500-6-77

**Published:** 2013-03-04

**Authors:** Yuan Wu, Jing Wang, Wenge Li, Hongbin Jia, Jie Che, Jinxing Lu, Lanzheng Liu, Ying Cheng

**Affiliations:** 1 Department of Hospital Acquired Infection Control and Prevention, National, Institute for Communicable Disease Control and Prevention, Chinese Center for Disease Control and Prevention, State Key Laboratory for Infectious Disease Prevention and Control, Beijing, China; 2 China-Japan Friendship Hospital, Beijing, China; 3 Municipal Centre for Disease Control and Prevention, Jinan, Shandong, China

**Keywords:** *Pichia fabianii*, Fungemia, Infant infection

## Abstract

**Background:**

Invasive fungal infections caused by uncommon fungi have increased in recent years. Hospitalized low-birth-weight infants are at high risk for neonatal fungal infections. *Pichia fabianii* is a rare pathogen causing blood infection, which has reportedly caused only 4 cases of fungemia and 1 case of endocarditis worldwide. Here, we describe the first case of a *P*. *fabianii* blood infection in a premature infant in China.

**Case presentation:**

On July 28^th^, a low-birth-weight (LBW, 1760 g) female infant born at 33^+4^ weeks of gestation was admitted to the pediatric intensive care unit with mild neonatal asphyxia. Until August 2^nd^, a mechanical respirator was used to assist respiration under the Continuous Positive Airway Pressure (CPAP) model. The baby had an increased body temperature and a fever. To prevent infection, Ceftriaxone Sodium (CS) was administered intravenously for three days, after which Cefepime was administered until August 13^th^. Chest X-rays showed suspected plaque-like shadows in the right lung. Blood cultures twice tested positive for fungal infection caused by *Candida pelliculosa* (recognized as *Pichia fabianii* later), which is first mis-identified by commercial kit. Hence, intravenous fluconazole was administered. However, cultures of other body fluids (e.g., urine, feces and sputum) tested negative for fungal infection. Routine tests and biochemistry of cerebrospinal fluid (CSF) were normal. Latex agglutination of *Cryptococcus neoformans* and fungi cultures in the CSF were also negative. After 14 days of intravenous fluconazole, blood was re-cultured, the result of which was negative. On August 30^th^, intravenous fluconazole was suspended. On Sep 3^rd^, the infant left the hospital in good health.

**Conclusions:**

This is the first case of a blood infection caused by *P*. *fabianii* in a LBW premature female infant in China. Risk factors for fungal infection include premature birth, as well as mechanical invasive operation and antibacterial drug usage. Whether such risk factors necessitate prophylactic use of antifungal drugs is an important question that has yet to be fully addressed. Additionally, the pathogen *P*. *fabianii* collected in this study was resistant to amphotericin B (AMB) and itraconazole (ITR). With the exception of the azole-resistant endocarditis case, all other cases have not demonstrated such a resistance. Finally, commercial biochemical methods used in routine practice are limited in their ability to identify *P*. *fabianii*. Molecular genetic based methods are imperative for identification of uncommon fungal species from disseminated infections.

## Background

Fungal infections are an increasing threat for: hospitalized patients using catheters (especially vascular), the immunocompromised (e.g., patients with cancer or HIV), premature neonates, and the elderly [1,2]. Low birth weight (LBW, <2500 g) is particularly neonates have an increased risk of fungal infection. Reports of invasive infections caused by uncommon fungi have increased in recent years [3,4]. Pichia, teleomorph stages of several *Candida* species, is an ascomycetous yeast species rarely involved in human infections [5]. Here we describe the first case of a fungemia caused by *Pichia fabianii* (also termed as *Candida fabianii* or *Lindnera fabianii*) in a premature female neonate in Beijing, China.

## Case presentation

A female infant, born at 33^+4^ weeks of gestation, was admitted to the pediatric intensive care unit at a general hospital in Beijing on July 28, 2010. She was diagnosed as premature and LBW (1760 g), with mild neonatal asphyxia. The mother was an elderly primipara (33-years-old), with a hysteromyoma, who had developed gestational hypertension. The mother had no history of infectious or other underlying disease. Due to low implantation of the placenta in the mother’s uterus, the baby had been delivered via caesarean. The infant was kept in a premature incubator (33-35°C), and was given multi-function intensive care, as well as prohibition of food and drink for 20 h, proper intravenous fluids according to need, and peripheral venous hyperalimentation. A respirator was used to assist breathing until August 2^nd^. To prevent infection, CS was administered intravenously (70 mg Qd) for three days, at which point cefepime was used instead. On August 5^th^, the incubator was aborted and inspired oxygen was given. On August 11^th^, the infant had a fever of 37.7°C, cold hands and feet, and a heart rate of 160-180 bpm; blood cultures were tested and cefepime was added to 140 mg Qd. On August 12^th^, chest X-Rays revealed plaque-like shadows in the right lung. Blood, urine, feces and sputum specimens were collected and sent for culturing and further identification. Blood cultures indicated fungal infection *(Candida pelliculosa)*, though all other bodily fluids tested negative in this respect. On August 13^th^, intravenous fluconazole (10 mg per day, micro pump 5 ml/h) treatment was begun and Cefepime treatment was aborted. The following day, the baby’s body temperature returned to normal and her heart rate was 148 bpm. The urine smear test was still negative, and blood was collected for re-examination. After treatment with intravenous fluconazole for 10 days, blood was retested. A lumbar puncture was performed to get the CSF, the routine test (color, clarity, TP, Glu and CI) and biochemistry of which was normal. A latex agglutination test of *Cryptococcus neoformans* and a fungi culture of the CSF were both negative. No abnormal symptoms were found by abdominal examination. On August 26^th^, blood cultures came back negative. On August 30^th^, 18 days after it had been begun, intravenous fluconazole administration was stopped. On September 3^rd^, the infant was released from the hospital in good health.

A drug sensitivity test was performed using ATB Fungus3 (BioMerieux, France). The MIC of this pathogen to 5 systemic antifungals was as follows: 5-flucytosine, 5FC (<4), amphotericin B, AMB (=1), fluconazole, FCA (≤1), itraconazole, ITR (=2) and voriconazole, VRC (=0.125), according to the manufacture’s procedure. The strain was sensitive to 5FC, FCA and VRC, but resistant to ITR, S/R to AMB following the interpretation of the susceptibility test. The yeast was sent to the laboratory in Chinese Center for Disease Control and Prevention (China CDC), and was cultured on Sabouraud’s Dextrose Agar, SDA (OXOID, UK) at 25°C for 48 h. A single white colony was picked and subcultured on a CHROMagarCandida plate (CHROMagar, France) at 30°C for 48 h, at which point the pink-white colonies were visible. This organism was capable of growing at 37°C and 42°C. Carbohydrate fermentations and assimilations of the pathogen, performed using API 20C AUX (BioMerieux, France), indicated high scores for *C*. *pelliculosa* infection. Germ tube tests of this organism, by inoculating several colonies into a bovine serum and incubating the suspension at 37°C for 2 h, were negative. After inoculation on meal agar with Tween 80 at 28°C for 24 h, pseudohyphae and budding yeast could be seen. The 26S ribosomal DNA and internal transcribed spacer (ITS) of this organism was amplified, sequenced and blasted, showing high similarity to *P*. *fabianii*. The universal primer pairs ITS1 (TCCGTAGGTGAACCT GCG G) and ITS4 (TCCTCCGCTTATTGATATGC), NL (GCATATCAATAAGCGGAGGAAAAG) and NL-4 (GGTCCGTGTTTCAAGACGG) were used to amplify these two DNA fragments [6]. The GenBank accession numbers of ITS and 26S were JQ342083 and JQ342084, respectively. MICs for AMB and ITR were re-determined by micro-dilution methods, as described by the Clinical and Laboratory Standards Institute (CLSI) [7] of China’s CDC. Results showed that this pathogen was AMB and ITR resistant, coinciding with results from the commercial kit.

## Conclusions

In this report, we are the first to describe the isolation of *P*. *fabianii* from the blood culture of a LBW infant in a Neonate Intensive Care Unit (NICU) in China. To our knowledge, only two cases of fungemia caused by *P*. *fabianii* in a preterm neonate have been reported worldwide (one in the USA [3] and one in France [5]). In the first case, the 5-week-old female neonate was born at 25^+3^ weeks of gestation to a healthy, 33-year-old mother [3]. This baby was given multiple courses of antibacterial antibiotics, mechanical ventilation and central catheter nutrition for her severe constitutional symptoms [3]. In the second case, the female infant was born at 24 weeks of gestation with an extremely low birth weight (ELBW, <1000 g) [5]. Central venous catheters were placed and cefotaxime and amikacin were used intravenously for a week in order to remove bacteria from bodily fluids [5]. In our case, the patient was also a premature baby girl, born at 33^+4^ weeks of gestation, with a LBW (1760 g). A respirator was applied to treat the neonatal asphyxia and antibacterial drugs were given for elevated body temperature and presumed infection. All three cases of neonate fungemia caused by *P. fabianii* occurred in a premature female infant, who had experienced invasive operation, and was treated with antibacterial drugs; all of these common characteristics are incidentally risk factors for fungal infection. This begs the questions: Is antifungal therapy necessary when these risk factors exist? In one case, mechanical operations were terminated when the fungal infection appeared and AMB was used successfully. Whether these behaviors are more important for the recovery of the patient is unknown [3].

Three other human infection cases have been reported [1,2,4], all of which were in adults: a 40-year-old male, a 46-year-old male, and a 53-year-old female. *P*. *fabianii* caused endocarditis in the first patient and fungemia in the other two. In these previously reported 5 cases (2 children, 3 adult), the organism was susceptible to AMB, 5FC and FCA, except for in the endocarditis case, in which the pathogen developed in vitro resistance to azoles in the course of antifungal treatment. In our case, *P*. *fabianii*, isolated from the blood before an antifungal was used, showed resistance to ITR and AMB. The molecular explanation for this strain’s different antifungal susceptibility necessitates further study.

Our case further allows us to study *P*. *fabianii* and its ability to cause invasive infection. NICU infants are a population at risk for developing invasive candidiasis [8]. Since *P*. *fabianii* has not been included in widely used commercial yeast diagnostic kits (API 20C AUX, ID32C and Vitek-2), it is difficult for clinicians to properly identify and thus has been commonly misidentified as *P*. *anomala* or *C*. *utilis*[9] only using commercial kit. In this study, the pathogen was initially identified as *C*. *pelliculosa* by API 20C AUX kit. And according to the molecular method mentioned in previous reports [1-4], the sequences of ITS region and D1/D2 variable region of the 26S rDNA were sequenced and BLAST in GenBank. The pathogen was subsequently identified as *P*. *fabianii*.

In summary, we report the first case of blood infection caused by *P*. *fabianii* of a LBW premature female infant in China. Because preterm birth, mechanical invasive operation, and usage of antibacterial drugs are risk factors for fungal infection, the prophylactic use of antifungal drugs should be considered under such risk factors. The pathogen *P*. *fabianii* collected in this study was resistant to AMB and ITR; this resistance was not present in any other cases of *P*. *fabianii* except the endocarditis case, in which the pathogen was resistant to azoles [3]. Molecular genetic based methods are imperative for future identification of uncommon fungal species from disseminated infections.

## Consent

Written informed consent was obtained from the patient’s guardian for publication of this case report and any accompanying images. A copy of the written consent is available for review by the Editor-in-Chief of this journal.

## Competing interests

The authors declare that they have no competing interests.

## Authors’ contributions

YW performed and interpreted all mentioned culture methods and molecular identification methods of the pathogen, and wrote the manuscript. JW and HBJ did the clinical identification of the pathogen, and collect the materials of the patient. WGL and JC took part in the culture of the pathogen. LZL and YC revised the manuscript. All authors read and approved the final manuscript.
